# Non-Destructive Detection of Different Pesticide Residues on the Surface of Hami Melon Classification Based on tHBA-ELM Algorithm and SWIR Hyperspectral Imaging

**DOI:** 10.3390/foods12091773

**Published:** 2023-04-25

**Authors:** Yating Hu, Benxue Ma, Huting Wang, Yujie Li, Yuanjia Zhang, Guowei Yu

**Affiliations:** 1 College of Mechanical and Electrical Engineering, Shihezi University, Shihezi 832003, China; hyt_0207@163.com (Y.H.); xgb@shzu.edu.cn (H.W.); lyj_shzdx@163.com (Y.L.); zyj_shz@163.com (Y.Z.); ygw986119533@163.com (G.Y.); 2 Key Laboratory of Northwest Agricultural Equipment, Ministry of Agriculture and Rural Affairs, Shihezi 832003, China; 3 Engineering Research Center for Production Mechanization of Oasis Characteristic Cash Crop, Ministry of Education, Shihezi 832003, China

**Keywords:** pesticide residues, SWIR hyperspectral imaging, metaheuristic optimization, machine learning, non-destructive detection

## Abstract

In the field of safety detection of fruits and vegetables, how to conduct non-destructive detection of pesticide residues is still a pressing problem to be solved. In response to the high cost and destructive nature of existing chemical detection methods, this study explored the potential of identifying different pesticide residues on Hami melon by short-wave infrared (SWIR) (spectral range of 1000–2500 nm) hyperspectral imaging (HSI) technology combined with machine learning. Firstly, the classification effects of classical classification models, namely extreme learning machine (ELM), support vector machine (SVM), and partial least squares discriminant analysis (PLS-DA) on pesticide residues on Hami melon were compared, ELM was selected as the benchmark model for subsequent optimization. Then, the effects of different preprocessing treatments on ELM were compared and analyzed to determine the most suitable spectral preprocessing treatment. The ELM model optimized by Honey Badger Algorithm (HBA) with adaptive t-distribution mutation strategy (tHBA-ELM) was proposed to improve the detection accuracy for the detection of pesticide residues on Hami melon. The primitive HBA algorithm was optimized by using adaptive t-distribution, which improved the structure of the population and increased the convergence speed. Compared the classification results of tHBA-ELM with HBA-ELM and ELM model optimized by genetic algorithm (GA-ELM), the tHBA-ELM model can accurately identify whether there were pesticide residues and different types of pesticides. The accuracy, precision, sensitivity, and F1-score of the test set was 93.50%, 93.73%, 93.50%, and 0.9355, respectively. Metaheuristic optimization algorithms can improve the classification performance of classical machine learning classification models. Among all the models, the performance of tHBA-ELM was satisfactory. The results indicated that SWIR-HSI coupled with tHBA-ELM can be used for the non-destructive detection of pesticide residues on Hami melon, which provided the theoretical basis and technical reference for the detection of pesticide residues in other fruits and vegetables.

## 1. Introduction

Hami melon is a kind of thick-skinned melon with delicious taste and rich nutritional value, which is a national geographical indication product. Hami melon is susceptible to powdery mildew, leaf blight, downy mildew, aphids, and other diseases during planting [1]. In order to prevent the above diseases, Hami melon growers often use Acetamiprid, Difenoconazole, Chlorpyrifos, and other pesticides for prevention and control [2]. However, in agricultural production, pesticides are often overused intentionally or unintentionally, which not only seriously threatens human health, but also causes environmental damage. Therefore, it is necessary to accurately detect pesticide residues on Hami melon.

The Food and Agriculture Organization of the United Nations (FAO) defines pesticides as substances or mixtures used to prevent, eliminate, or control harmful organisms that harm agriculture, as well as to purposefully regulate, control, and affect the metabolism, growth, and other processes of plants and harmful organisms [3]. The growing population has put forward a stronger demand for food, and the pesticide market has also increased significantly. With the increasing awareness of consumer health, people are increasingly focusing on food safety, more and more researchers begin to pay attention to the detection of pesticide residues in fruits [4]. In previous studies, techniques such as supercritical fluid chromatography (SFC), immunoassay method, liquid chromatography (LC), and gas chromatography-mass spectrometry (GC-MS) have been widely applied in the field of pesticide residue detection [5,6,7,8]. Although these traditional methods had high detection accuracy and sensitivity, they were time-consuming, costly, complex in operation, and dependent on many chemical reagents, which were wasteful and polluting, and were not conducive to popularization. What’s more, traditional chemical detection methods are destructive, and the detected Hami melon could not be sold. Therefore, it is essential and significant to find a rapid and non-destructive method to detect pesticide residues on Hami melon.

Scholars have conducted related research to search the capacity of different non-destructive detection technologies, such as machine vision, electronic nose, acoustic technology, and spectral technology, for food quality assessment [9,10,11]. Among them, the spectroscopic technology depending on simple and non-destructive methods have attracted great attention [12]. Spectroscopic techniques, including near infrared spectroscopy, fluorescence spectroscopy, Raman spectroscopy, and terahertz time-domain spectroscopy, have been proved to be useful for the measurement of pesticide residues in food [13,14,15,16,17]. However, one of the main disadvantages of these spectral analysis methods is that the spectral data are obtained from a single point (or regional area). Although there is no destruction or preprocessing, the point source sampling method limits the integrity of collected data and cannot ensure that the collected data can completely and accurately express the required information, which easily leads to unsatisfactory pesticide residue detection effect.

In contrast, hyperspectral imaging (HSI) is considered as a potential technology to solve the above-mentioned defects [18,19]. HSI is a rapidly developing non-destructive testing technology, which has been gradually applied to agricultural products testing in recent years [20,21]. It has the advantages of no pollution, no damage, automation, high efficiency and so on. The spatial distribution spectral information at each pixel of the object can be obtained. The spectral and image information of the sample can be obtained simultaneously [22]. At present, the research regarding HSI technology in agricultural product quality detection is relatively mature [23].

Some scholars have used HSI to discriminate and assess the quality and safety of diverse foods, and obtained satisfactory results, such as defect detection of citrus [24], classification of mildew, health, and damage of peanuts [25], prediction of potential pest infection of apples [26], and identification of Cucumber Green Mottle Mosaic Virus (CGMMV) infection in watermelon seeds [27]. HSI technology has also been extensively employed in the study of pesticide residues. Sun et al. studied chlorpyrifos residues in mulberry leaves at different concentrations and combined hyperspectral imaging technology with SVR prediction model, obtaining good results with root mean square error (RMSE) and determination coefficient (R^2^) of prediction set of 27.719 and 0.874, respectively [28]. Relevant studies showed that HSI technology can be applied to identify pesticide residues in lettuce. Two models, CARS-SPA-LSSVR and RF-RFE-SPA-LSSVR, were used to detect fenvalerate and dimethoate, respectively, and good results were obtained [29]. Some scholars also applied this technology to detect the contamination of chlorpyrifos and imidacloprid in edible jujube fruits, and achieved good results by using the ES-AWLSGSD-RC-LWPLSR model constructed by using only eight feature wavelengths [30]. However, these studies mainly used HSI in the spectral range of 400–1000 nm or 900–1700 nm to assess food quality and safety. Any substance has energy and produces unique light waves. Therefore, the light waves reflected by different substances are not the same. In recent years, the use of short-wave infrared (SWIR) spectral range (1000–2500 nm) has been gradually favored by researchers, such as the classification of apple potential scratches [31], the identification of peanut kernel mildew [32], and the rapid prediction of the water content of single cucumber seed [33] and the soluble solid content of Hami dried jujube [21]. Some studies have proved that the discovery of this spectral region was affected by the vibration and overtones of the main structural components of organic molecules [34]. Most pesticides are organic compounds and contain more organic molecules. So, the SWIR spectral range may be more suitable for the discrimination of pesticide residues on fruits. However, there are few studies using HSI to detect pesticide residues on thick-skinned fruits in the SWIR spectral range. Therefore, one of the objectives of this study is to investigate the possibilities of using SWIR-HSI for the accurate detection of pesticide residues on the surface of Hami melon.

The collected original spectrum often contains the random noise of the instrument. In addition, the introduction of baseline drift, light stray scattering, and other information will also affect the corresponding relationship between the spectrum information and the indicators to be measured, thus affecting the stability of the model. Therefore, the preprocessing of spectral data is a key step before modeling and analysis [35]. Preprocessing treatments were commonly used in spectral analysis, such as Savitzky–Golay smoothing (SGS), multiple scatter correction (MSC), and standard normal variate transformation (SNV), etc. These treatments were widely used in hyperspectral imaging to determine the quality of agricultural products [36,37]. Extreme learning machine (ELM), support vector machine (SVM), and partial least squares discriminant analysis (PLS-DA), three classical machine learning classification models, have been widely used in HSI field because of their simple classification ideas, strong learning ability, and good classification effect [38,39,40]. Considering that the model accuracy of classical machine learning model is affected by model parameters, data volume, and other factors, resulting in problems such as poor model stability and generalization, it is very important to optimize the classical classification model. The metaheuristic algorithm has many advantages, such as faster optimization speed, more effective search for the global optimal solution of complex optimization problems, and stronger stability and adaptability. The most typical problem dealt with by the metaheuristic algorithm is optimization. Therefore, some researchers tried to use the metaheuristic algorithm to adjust the traditional model, and it has been successfully applied in bacterial food-borne pathogen classification, pesticide residues in lettuce, rice mold colony detection, and other fields [41,42,43].

Therefore, the objective of this study was to apply HSI technology combined with the ELM model optimized by the metaheuristic algorithm to identify a single class of pesticide residues (None, Acetamiprid, Malathion, Difenoconazole and Beta-cypermethrin). The main objectives of this study were: (1) to explore the feasibility of HSI technology based on SWIR spectral region (1000–2500 nm) for pesticide residues on Hami melon; (2) to assess the effectiveness of different preprocessing treatments for spectral data analysis; (3) to develop an ELM optimized by the adaptive t-distribution honey badger algorithm (tHBA) for identifying pesticide residues; and (4) to compare the optimization effects of different metaheuristic algorithms and the classification effects of the established models.

## 2. Materials and Methods

### 2.1. Samples Preparation

Four standard pesticides were purchased from a local agricultural raw material market in Shihezi, Xinjiang, China. Acetamiprid (Active ingredient content 70%, water dispersible granule, Shandong Baixin Biotechnology Co., Ltd., Jinan, China), Malathion (Active ingredient 70%, emulsifiable oil, Ningbo Sanjiang Yinong Chemical Co., LTD., Ningbo, China), Difenoconazole (Active ingredient 20%, microemulsion, Chengdu Kelilong Biochemical Co., Ltd., Chengdu, China), and Beta-cypermethrin (Active ingredient 4.5%, emulsion, Jiangsu Yixing Xingnong Chemical Products Co., Ltd., Yixing, China) were used as research objects.

Hami melons (Xizhoumi No.25) were purchased from the local agricultural products trading center in Shihezi, Xinjiang, China, in August 2021. All samples were oval and weighed approximately 3-4 kg. Before collecting data, all Hami melons samples were numbered after wiping, and then reposed in a well-ventilated room at 22 °C and 40% relative humidity for 24 h to avoid being affected by the external environment. The 200 samples were arbitrarily selected and separated into five equal groups (40 samples per group). Then, Acetamiprid, Malathion, Difenoconazole, and Beta-cypermethrin were mixed with distilled water respectively to prepare 1:1000 pesticide solution, which was evenly sprayed on the surface of Hami melon, and recorded as group 1, group 2, group 3, and group 4. In addition, the remaining 40 Hami melon samples were used as the control group, and samples were homogeneously sprayed with distilled water, which was recorded as group 0. In the end, every sample was deposited indoors for 12 h.

### 2.2. Hyperspectral Imaging Acquisition and ROI Spectrum Acquisition

#### 2.2.1. Hyperspectral Imaging System

**Figure 1:**
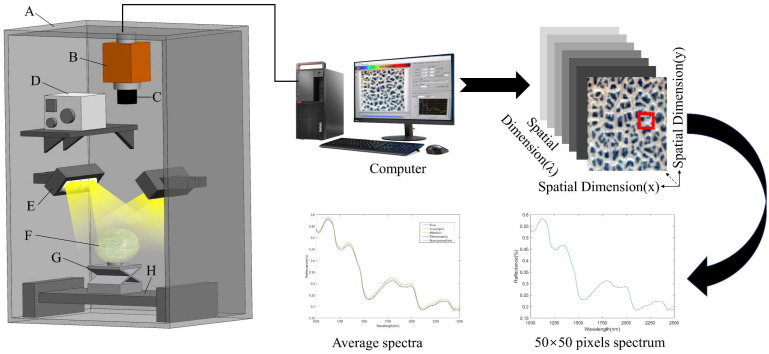
SWIR-HSI acquisition system and three-dimensional (3D) data cube. (A) Box with black inner surface; (B) Imaging Spectrograph; (C) Lens; (D) Light source regulator; (E) Halogen lamps; (F) Sample of Hami melon; (G) Lifting platform; (H) Electric moving stage.

The SWIR hyperspectral imaging system consists of an imaging spectrometer (ImSpector N25E2⁄3^’’, Specim, Oulu, Finland), a high-resolution camera (Zephir-2.5–320, Photon Etc., Montreal, QC, Canada), two 150 W halogen surface light sources adjusted at an angle of about 45° to illuminate the camera’s field of view, an electrically positioned sample stand operated by a stepper motor, a computer equipped with data acquisition software (Spectral Image System, Isuzu Optics Corp., Taiwan, China), and a box with black inner surface. Hyperspectral images of Hami melon samples containing pesticide residues were obtained in diffuse reflection mode, and the spectral range was 1000–2500 nm. The measurement was carried out in a dark room to avoid the interference of stray light. In addition, the SWIR hyperspectral imaging system is shown in Figure 1.

#### 2.2.2. Imaging Acquisition and Calibration

The instrument was preheated for 30 min before use, and the samples of Hami melon were collected on the electric moving stage. Proper adjustment of parameters was crucial for gaining perspicuous and distortion-free hyperspectral images. In this study, the correlation coefficient of HSI was set as follows. The exposure time of the camera was adjusted to 4.1 ms, and the speed of the electric mobile stage was adjusted to 53.4 mm/s. As the electric moving stage moved, the acquisition system simultaneously acquired the image and spectral information of the Hami melon, and repeated the operation until all the sample information was obtained. In fact, the directly obtained hyperspectral images of Hami melon need to be black and white corrected first and cannot be immediately used in experiments. When collecting hyperspectral images, because the illumination direction of the light source was fixed, the light intensity on the sample surface changed greatly, and the existence of camera dark current can bring noise to the spectral data. Whiteboard data and blackboard data are reference data for reflectivity calibration objects. By the white and dark references, the gap of data with different light intensities can be decreased, so that the relevant spectra can be compared and analyzed under different environments and light intensities. The white and dark references principle is shown in the equation below:

(1) $$R_{c}=\frac{R_{r}-R_{d}}{R_{w}-R_{d}}$$

where $R_{r}$ is the raw spectral value, $R_{w}$ is the all-white reference value, $R_{d}$ is the all-black reference value, and $R_{c}$ is the corrected value.

#### 2.2.3. ROI Spectrum Acquisition

The image resolution of the hyperspectral camera is 320 × 256 pixels, the spectral resolution is 6.20 nm. The spectral range is 982.38–2618.37 nm, which can collect hyperspectral images of 288 wavelengths. After edge band screening, the obtained band range was 1000–2500 nm, and the hyperspectral images of 233 wavelengths were retained. For each Hami melon, one hyperspectral image was collected every 90° rotation along the equatorial direction. So, 4 hyperspectral images can be collected from one Hami melon sample, and 800 hyperspectral images can be collected from 200 Hami melon samples. After the hyperspectral image information was collected, the hyperspectral image was imported into ENVI software, and pixel blocks of 50 × 50 at the equatorial position of Hami melon were randomly designated as regions of interest (ROI). A total of 800 average spectra were collected, and these spectra were stored for the establishment of classification model.

### 2.3. Spectral Data Preprocessing

Preprocessing can remove as much extraneous noisy data as possible that has an interfering effect on the predicted results. Spectral information is often mixed with unwanted noise and background interference, such as stray light and electrical noise, which should be eliminated or reduced, which is the goal of spectral preprocessing [44]. In this study, we applied and compared three commonly used treatments, including MSC, NM and SNV, respectively. The purpose of MSC is to compensate the decrease of signal-to-noise ratio caused by light scattering, and the spectral data obtained after scattering correction can effectively eliminate the spectral differences caused by different scattering levels [45]. NM uses scaling, panning and other operations to convert the data into a specific interval, thereby eliminating the influence of data dimension and making the data indicators comparable [46]. SNV can eliminate the differences in individual samples due to factors such as particle size and can be used to correct spectral errors caused by scattering between samples [47].

### 2.4. Establishment of tHBA-ELM Model

#### 2.4.1. Honey Badger Algorithm

Currently, more and more researchers are trying to introduce various numerical optimization methods into their algorithms, among which metaheuristic optimization algorithms have been developed substantially. Enlightened by the foraging habits of honey badgers in nature, a more intelligent metaheuristic optimization algorithm, the honey badger algorithm (HBA) [48], has been proposed. This algorithm developed an effective search strategy for solving optimization problems in mathematics. In the HBA algorithm, HBA imitates the foraging behavior of badgers in nature and divides optimization into two modes: “digging” and “honey”. In the digging mode, the badger mainly depends on the odor intensity of the target, estimates the position of the target according to the odor, and selects the appropriate position near the target for digging. In the honey mode, the badger mainly locates the target location directly according to the location of the honeyguide bird. HBA is effective in solving optimization problems with complex search space and has advantages in convergence speed and exploration-development balance. It has strong search ability and fast convergence speed.

The HBA algorithm is simple in design and clear in thought. The algorithm mainly consists of steps such as population initialization, fitness assessment, and honey attraction calculation, providing good local search capability based on honey attraction. In addition, the density factor is set to ensure the global searching ability of the algorithm. Through testing, the algorithm showed great properties in solving most test functions.

Compared with the common metaheuristic algorithm, the algorithm has the advantages of simple mechanism, less parameters, fast convergence speed, and has advantages in convergence speed and exploration and development balance. Therefore, this study intended to use HBA to optimize the parameters and optimize the classification model to improve the classification accuracy.

#### 2.4.2. Honey Badger Algorithm Based on Adaptive t-Distribution Mutation Strategy

Aiming at the issue that the metaheuristic algorithm tends to become trapped in local convergence, mutation operation is a common method used to leap out of local convergence. Introducing mutation operator is beneficial to further improve the ability of the algorithm to find the optimal solution. Because the introduction of mutation operator can make the algorithm have a certain local random search ability, on the one hand, it accelerates the convergence to the optimal solution in the late stage of solution, and on the other hand, it also maintains the diversity of solutions. The iteration times was regarded as the degrees of freedom parameter of t-distribution, and the adaptive t-distribution mutation method was used to disturb the individual position to improve the algorithm’s ability [49]. This method was called tHBA.

Gaussian distribution (GD) and Cauchy distribution (CD) have been proven to effectively lift the optimization capability of the algorithm. GD can enhance the searching performance of individuals near the optimal point and accelerate the convergence speed of the algorithm. CD can enhance the search ability of individuals in the solution space and increase the diversity of the population, while t-distribution has the advantages of both GD and CD. As the iteration of the algorithm begins, the t-distribution gradually changes from similar to CD to similar to GD as the number of iterations increases. Therefore, the adaptive t-distribution mutation method was used to disturb the individual position and improve the ability of the algorithm to jump out of the local optimum. In this study, the t-distribution mutation operator with the degree of freedom parameter of iter as t-distribution is used to disturb the position of the badger, so that the algorithm has better global development capability in the early iteration and has better local exploration ability in the later iteration, and enhances the convergence speed of the algorithm. Specific location updates are as follows:

(2) $$X_{i}^{t+1}=X_{i}^{t}+X_{i}^{t}\cdot t\left(iter\right)$$

where, $X_{i}^{t+1}$ is the position of the disturbed badger, and $X_{i}^{t}$ is the position of the badger i at the t iteration. The proposed update equation adds a random interference term $X_{i}^{t}\cdot t\left(iter\right)$ on the basis of $X_{i}^{t}$, which helps the algorithm leap out of the local optimum by introducing random disturbance information on the basis of the current position information. With the number of iterations iter increased, t-distribution gradually draws close to the Gaussian distribution, which is useful to enhance the convergence speed. The addition of adaptive t-distribution mutation operator effectively enhances the property optimization of the algorithm and avoids local optimization.

**Figure 2:**
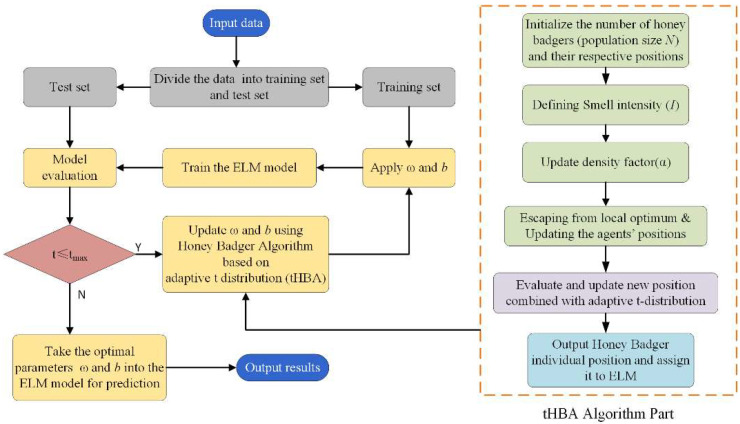
tHBA-ELM Algorithm Flow Chart.

The algorithm flow of tHBA is as follows, the flow chart for which is shown in the tHBA Algorithm part in Figure 2:

(1) Determine the tHBA algorithm parameters (population size N, mutation ratio, etc.), using Equation (3) to initialize the population.
(2) Define smell intensity of prey I. Strength and prey concentration are related to the distance between it and the badger. If the smell intensity is strong, the exercise speed is higher, and the reverse is also true, as defined by Equation (4).
(3) Update density factor (α). The value of the α decays with the increase in the number of iterations to ensure the stability of the overall environment when the algorithm transitions from the exploration state to the exploitation state, as in Equation (5).
(4) Escape from local optimum and update the agents’ positions. HBA uses a sign F (F: is a sign to change the agent’s search direction.) to change the search direction to take advantage of the high opportunity for agents to scan the search space strictly. The HBA location update process is separated into two modes: “digging” and “honey”.
(5) Generate the random number rand between the interval of 0 to 1. If rand < P (P: mutation probability) then according to Equation (2) adaptive t-distribution mutation, calculate the fitness value and update the badger position.
(6) When the number of iterations exceeds $t_{max}$, the optimization process will terminate and output the global optimal position, namely the optimal values of ω (connection weight between input layer and hidden layer) and b (neuron threshold of hidden layer) in ELM.

(3) $$x_{i}=lb_{i}+r_{1}\times \left(ub_{i}-lb_{i}\right)$$

where $x_{i}$ is the coordinate of the ith badger individual, $lb_{i}$ and $ub_{i}$ are the upper and lower boundaries of the optimization space, and $r_{1}$ is a random variable in the interval of 0 to 1.

(4) $$\left\{\begin{array}{c} I_{i}=r_{2}\times \frac{S}{4\pi d_{i}^{2}}\\ S={\left(x_{i}-x_{i+1}\right)}^{2}\\ d_{i}=x_{Prey}-x_{i} \end{array}\right.$$

where $r_{2}$ is a random variable in the interval of 0 to 1, S is the aggregation degree of honey, and $d_{i}$ is the distance between the badger and honey.

(5) $$\alpha =C\times exp\left(\frac{-t}{t_{max}}\right)$$

where $t_{max}$ is the maximum number of iterations, C is a constant≥1 (default=2).

#### 2.4.3. ELM Classification Model Based on tHBA Optimization

ELM is a simple and reliable single hidden layer feedforward neural network learning algorithm. The improved neural network structure improves the learning speed and got a wide application. ELM is mainly composed of the input layer, output layer, and hidden layer in the middle. ELM only needs to set the appropriate number of hidden layer nodes, arbitrarily produce all the parameters required for the hidden layer, and finally use the least square method to determine the weight of the output layer. ELM possesses the advantages of low training complexity, high learning efficiency for targets, and high accuracy after generalization in comparison with traditional neural networks. In this study, the tHBA algorithm was used to search for optimal ω and b in ELM. The flow chart is shown in Figure 2.

The procedure of the tHBA-ELM algorithm was as follows:

(1) Input the preprocessed spectral data of Hami melon samples and divide the data into training set and test set according to the ratio of 3:1, with 600 spectra in training set and 200 spectra in test set. Then the training set was used as the training sample of tHBA-ELM model, and the test set was used as the model verification.
(2) The position of the badger was updated by tHBA and assigned to ω and b in ELM. The ELM model was trained by using the ω and b obtained from each update.
(3) Set the number of iterations to 300. When the number of iterations reached 300, tHBA output the best fitness value corresponding to the badger position, using the best ω and b to establish the best ELM model, and output the model results.

### 2.5. Assessment Standard of Models

To evaluate and compare the effect of the model, four evaluation indexes (accuracy, precision, sensitivity, and F1-score) were selected to represent the performance of the model. Accuracy refers to the percentage of discriminated correct results in the total sample; precision refers to the probability of actually positive samples among all the discriminated positive samples; sensitivity refers to the probability of being discriminated as a positive sample among the actually positive samples. The F1-score combines the performance of precision and sensitivity. To find a balance between the two, an F1-score appears. These evaluation parameters are calculated as follows:

(6) $$Accuracy=\frac{\left(TP+TN\right)}{\left(TP+FP+TN+FN\right)}$$

(7) $$Precision=\frac{TP}{TP+FP}$$

(8) $$Sensitivity=\frac{TP}{TP+FN}$$

(9) $$F1\text{-}\mathrm{score}=\frac{2\times Precision\times Sensitivity}{Precision+Sensitivity}$$

Among them, TP: true positive, positive samples are classified as positive samples. FP: false positive, negative samples are classified as positive samples. TN: true negative, negative samples are classified as negative samples. FN: false negative, positive samples are classified as negative samples.

## 3. Results

### 3.1. Spectral Characteristics of Pesticide Residues

**Figure 3:**
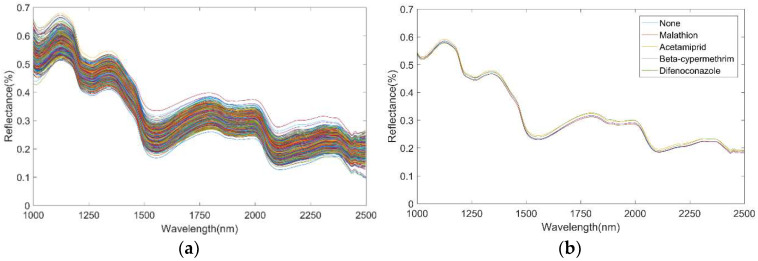
Raw spectra and average reflectance spectra. (**a**) raw spectra; (**b**) average reflectance spectra.3.2 Establishment and analysis of classical machine learning classification model.

The original and average diffuse reflectance spectra of different pesticide residues on Hami melon are shown in Figure 3. Figure 3a shows the original diffuse spectra and Figure 3b the average diffuse reflectance spectra. It can be observed from Figure 3 that the hyperspectral reflectance of various pesticide residues was varied, but the variation tendency of spectral curves was parallel. Specifically, there were relatively obvious spectral differences between the samples with different kinds of pesticide residues and the samples without residue in the range of 1493–2038 nm. Among them, the reflectance of Hami melon samples without residues in the range of 1493–1663 nm was the lowest, the reflectance of Hami melon samples sprayed with pesticides was higher than that of no residue samples, and the reflectance of Hami melon samples sprayed with Malathion was the highest. There were characteristic peaks and valleys in the specific spectral range, six peaks (1125, 1340, 1801, 2000, 2313, and 2370 nm) and six valleys (1020, 1269, 1552, 1929, 2432, and 2466 nm) appeared at similar wavelengths. These absorption wavelengths were related to the periodic tensile vibrations of C-H, O-H, and N-H bonds, which were the most fundamental chemical bonds of organic compounds [50]. The 1020 nm was in the low reflectivity range with high absorbance, which corresponded to the second overtone of C-H tensile vibration [51]. Wavelengths around 1125 and 1269 nm can be designated as the second overtone of C–H stretching in carbohydrates [52]. The 1552 nm and 1340 nm were mainly related to the chlorophyll absorption band of Hami melon, and 1552 nm was also related to N-H first-order frequency doubling [53]. The absorption peaks of water were approximately 1801 nm and 1929 nm, which was also the first overtone and combination mode of O-H group stretching [54]. While the strongest reflection trough (1935–1952 nm) can be attributed to the combination of O-H stretching and O-H bending of water [55]. The peaks and valleys at 2000 nm, 2313 nm, 2370 nm, 2432 nm, and 2466 nm were related to the frequency synthesis of C-H and O-H groups [56]. However, the appearance of these peaks and valleys cannot accurately and directly represent the presence of specific components in the sample, as the reflectivity at each location represents a complex set of component information. Especially, there is overlap in the spectral information of organic pesticides. In this study, the implied relationship between spectral data and pesticide residues was explored and analyzed.

### 3.2. Establishment and Analysis of Classical Machine Learning Classification Model

SVM, PLS-DA, and ELM were commonly used machine learning classification models, which were widely used in hyperspectral. SVM model is a supervised learning method based on statistical learning theory. After mapping the input data to high-dimensional space, linear SVM is applied to obtain nonlinear SVM, which was widely used in pattern recognition. The PLS-DA method is a discriminative classification strategy based on PLS, which can decompose the spectrum and the category matrix concomitantly, enhance the function of category information in spectral decomposition, and maximizes the extraction of the differences between different categories of spectra. ELM is a simple and reliable single hidden layer feedforward neural network learning algorithm, which has the advantages of fast training speed and high efficiency during learning, and high accuracy of generalization model. It is widely applied in prediction, classification, discrimination, and other issues. In this section, three classical machine learning classification models were compared and analyzed, and the better model was selected. The experimental results are shown in the Table 1.

In the training of the model, RBF was selected as the kernel function of SVM, and the number of latent variables (LVs) with the smallest cross-validation error was selected to construct the PLS-DA model. The number of hidden layers of the ELM model was set to 135, and the kernel function was set as sig. The results showed that the three classical classification models can complete the classification task. Scholars in related fields also used machine learning model to complete classification tasks and obtain good results [23]. The accuracy of test sets of three classical classification models was 79.50%, 77.50%, and 75.50%, respectively.

Among these models, the performance of ELM classifier was significantly better than the other two. The precision value of ELM model was the highest, which was 80.29%. The precision values of SVM and PLS-DA were similar. The sensitivity of ELM model was 79.50%, which was 2.00% and 4.00% higher than that of SVM and PLS-DA models, respectively. There was sometimes a trade-off between precision and sensitivity. In order to achieve a balance between these two indicators, the F1-score indicator was selected, which was the harmonic average of the above two. Among them, the ELM model got the highest F1-score of 0.7962, the second was 0.7760 of the SVM model, and the lowest was 0.7555 of the PLS-DA model. The closer the F1-score value is to 1, the better the classification effect of the model is.

The performance test results of three different classical machine learning classification models showed that ELM had more advantages than SVM and PLS-DA, so ELM was selected to complete the subsequent model optimization.

### 3.3. Comparative Analysis of Spectral Preprocessing Treatments

Three types of preprocessing treatments (NM, MSC, and SNV) were used in this study to correct the original spectra. After preprocessing, the spectral data retained the absorption characteristics of 233 original spectra, and then ELM was used to model and analyze the spectral data after different preprocessing, which improved the classification ability of the model to a certain extent.

ELM classification models were established based on NM, MSC, SNV, and the intact spectrum without preprocessing. All three preprocessing treatments can enhance the classification accuracy of the model. Table 2 shows the model performance of the test set in terms of accuracy, precision, sensitivity, and F1-score. Compared with the untreated original spectrum, the ELM classification model developed based on the preprocessing spectral data showed better overall discrimination performance. Proper spectral preprocessing improved the recognition accuracy of the model. The classification effects of the MSC-ELM and SNV-ELM were similar, and the difference between them was less than 1.00% under various evaluation criteria. In contrast, when the NM-ELM model was used to classify different pesticide residues on the surface of Hami melon, in single classification, the accuracy of Acetamiprid was 90.00%, the precision was 92.31%, the sensitivity was 90.00%, and the F1-score was 0.9114. Compared with the ELM classification model constructed after MSC and SNV preprocessing, the classification effect of the NM-ELM model was better, showed higher accuracy (82.00%), and its precision, sensitivity, and F1-score were 82.07%, 82.00%, and 0.8201, respectively. So, the NM-ELM model was selected for further optimization.

### 3.4. Establishment and Analysis of tHBA-ELM Models

Since the parameters of ELM directly affect its classification accuracy, how to choose the optimal parameters is a problem that must be considered to improve the ELM classification model. The classification performance of ELM is mainly affected by the connection weight ω between the input layer and the hidden layer and the neuron threshold b of the hidden layer. The metaheuristic optimization algorithm is widely used in model parameter optimization [39,41,57]. In order to overcome this problem, the optimization technology of HBA was used to solve the selection and optimization of ω and b.

Two optimization strategies, GA and HBA, are used to optimize ELM. In order to compare the classification performance of the two optimization models, experiments were carried out under the same data set, and the results were shown in the Table 3. The number of hidden layers of GA-ELM and HBA-ELM models was set to 120, the population size was set to 50, and the number of iterations was set to 300. The initial input weight and hidden layer bias of the ELM model were optimized by GA and HBA algorithms. The discrimination accuracy of different pesticide residues in Hami melon in GA-ELM and HBA-ELM models were all superior to that in ELM model, which indicated that the classification accuracy of different pesticide residues on the surface of Hami melon in ELM model could be effectively improved by optimizing the parameters of ELM model through Metaheuristic algorithm. Other scholars have also used the combination of GA and machine learning model to achieve good results [58,59]. The accuracy of ELM classification model optimized by GA and HBA increased by 6.50% and 8.50%, precision by 6.65% and 8.59%, sensitivity by 6.50% and 8.50%, and F1-score by 0.0651 and 0.0850, respectively. It can be seen from the evaluation criteria of individual categories that the classification accuracy of HBA-ELM was higher than GA-ELM, and the F1-score of Beta-cypermethrin increased the most, indicating that the HBA-ELM model was more stable and accurate than the GA-ELM model.

For the sake of further improving the discrimination accuracy, the HBA-ELM model was optimized using adaptive t-distribution variation based on the above model. After many experiments, the coefficient of the mutation probability P was selected as 0.8. Compared with the HBA-ELM classification model, the evaluation criteria of the ELM model optimized by the tHBA were generally improved, with the overall accuracy of 93.50%, precision of 93.73%, sensitivity of 93.50%, and F1-score of 0.9355. Compared with the HBA-ELM, the discrimination accuracy of Acetamiprid and Difenoconazole in the individual categories reached 95.00%. The other three evaluation criteria of Acetamiprid were the highest among the five categories, precision was 97.44%, sensitivity was 95.00%, and F1-score was 0.9620.

**Figure 4:**
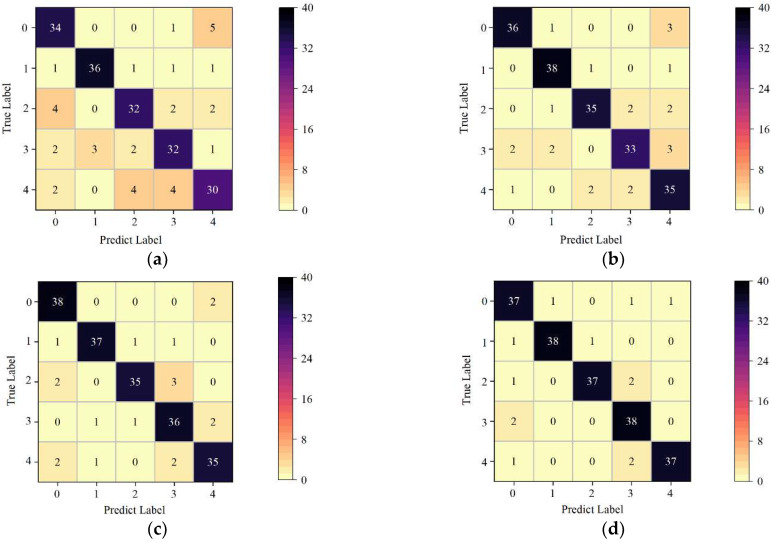
ELM and the improved ELM model confusion matrix. (**a**) NM-ELM; (**b**) NM-GA-ELM; (**c**) NM-HBA-ELM; (**d**) NM-tHBA-ELM.

In order to further explore the detailed classification performance of each level, Figure 4 shows the confusion matrix of the classification results of the four classification models, i.e., NM-ELM, NM-GA-ELM, NM-HBA-ELM, and NM-tHBA-ELM, established by 200 different spectra of the test set. In the Figure 4, 0 in the horizontal and vertical coordinates represented none, 1 represented Acetamiprid, 2 represented Malathion, 3 represented Difenoconazole, and 4 represented Beta-cypermethrin. In terms of overall recognition ability, the NM-tHBA-ELM model only misjudged 13 samples, which was the least misjudged among the four models, as shown in Figure 4d. Moreover, the number of misjudged types was basically the same. In the NM-ELM model, as shown in Figure 4a, the misjudgment degree of Beta-cypermethrin in NM-ELM model was high, with 10 samples being misjudged (misjudgment rate: 20.00%). The misjudgment degree of Acetamiprid was the lowest in the five categories, and the misjudgment rate of the other four categories was 2.50%. In Figure 4b, the identification effect of Acetamiprid samples in the NM-GA-ELM model was better, and only two samples were misjudged, followed by the misjudgment rate of no residual samples of 3.00%, and most of them were misjudged as Beta-cypermethrin. In the NM-HBA-ELM model, as shown in Figure 4c, the misjudgment rate of Malathion and Beta-cypermethrin was 12.50%, but the overall discriminant ability was higher than that of the original ELM classification model. The test results showed that the classification accuracy of tHBA-ELM was higher than ELM, GA-ELM and HBA-ELM models, and it had good classification accuracy for different types of pesticide residues, which can be used for the identification of pesticide residues on Hami melon surface. In contrast, when SWIR was used to detect pesticide residues, the classification effect in this study was slightly lower than that of garlic chive (*Allium tuberosum*) leaves. This may be because the proportion of pesticide solution in this study was low, and the types of pesticides were different. In addition, the samples used in this study were thick-skinned melons, which were quite different from the physicochemical properties of garlic chive (*Allium tuberosum*) leaves, which may also be the reason for the inconsistent results [51].

**Figure 5:**
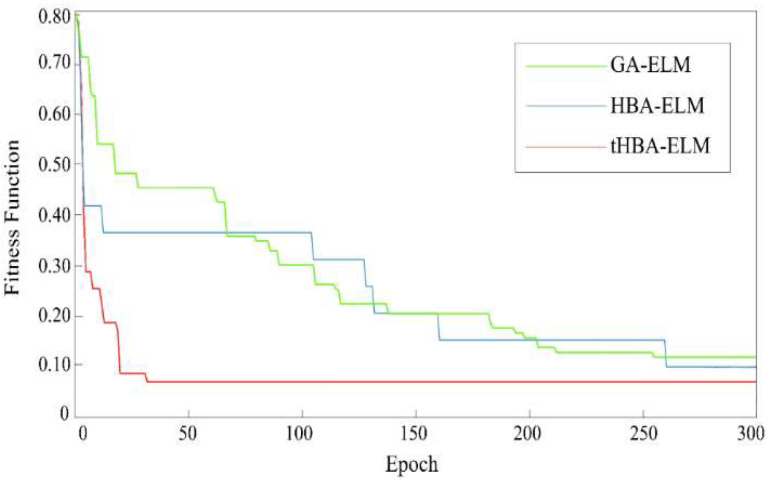
Optimization process of GA-ELM, HBA-ELM, and tHBA-ELM.

The fitness curves of GA-ELM, HBA-ELM, and tHBA-ELM classification models are shown in Figure 5. It can be observed from Figure 5 that tHBA-ELM had the fastest convergence speed and was completely convergent when the number of iterations was 30 generations. However, GA-ELM and HBA-ELM were still not completely convergent when the number of iterations was 250 generations. In addition, the fitness value of tHBA-ELM classification model (the fitness value of this model was the error rate of the test set) was lower than 0.1, while the fitness value of GA-ELM and HBA-ELM classification models were both higher than 0.1. In view of the possibility that HBA-ELM may fall into local convergence, an adaptive t-distribution was added to the HBA-ELM model, so that it could jump out of the local optimum and converge as soon as possible. It can be seen from the Figure 5 that tHBA-ELM had a fast convergence speed and the lowest fitness value.

## 4. Discussion

HSI is an effective method to realize the rapid and non-destructive detection of pesticide residues on Hami melon surface. In this study, SWIR-HSI was used to identify the pesticide residues on the surface of Hami melon. The high detection accuracy using the SWIR band may be due to the fact that the peaks and valleys of the spectral profile of this band are very distinct, or that the band has a higher reflectance for organic matter. At the same time, some scholars have used the band to detect pesticide residues in fruits and vegetables with good results [51].

When PLS-DA is used in multivariate modeling, if the dependent variables put into the model have little or no influence, the model structure will be unstable and even the discrimination results of the model will be poor. In addition, PLS-DA is a linear modeling method, SVM and ELM are both nonlinear modeling methods, and the spectral information of pesticide residue samples on the surface of Hami melon is nonlinear, which may also lead to poor classification effect of PLS-DA. In essence, both SVM and ELM map problems to a high-dimensional space, and then conduct classification research in the high-dimensional space. The classification effect is affected by the mapping method. If the mapping method is good, it will show obvious differences in high-dimensional space, and the classification effect will be good. If the mapping method is not shown in high-dimensional space, the classification effect will be poor. ELM is superior to SVM because it can be projected into high dimensions in infinite ways, and its training speed is very fast. We can adjust ELM many times and choose a better part from it. While with SVM, once the kernel function is determined, the mapping mode is uniquely determined, and its training speed is slower than ELM, so its performance is slightly worse than ELM. Similarly, some scholars have chosen spectroscopic techniques combined with ELM models for non-destructive detecting of the quality of agricultural products [60].

The acquisition of original hyperspectral data will be affected by the detection environment, operation level, instrument status, and other factors, so there will be more noise and interference in the original hyperspectral data. In order to minimize the impact of noise on data analysis and further explore and improve the classification performance of ELM, the original spectral data needs to be pretreated. The greatest improvement in model accuracy was achieved using the NM preprocessing method, and the model performance was also improved using MSC and SNV, and both improvements were similar. This may be because the purpose of MSC and SNV is basically the same, mainly to eliminate the scattering effect caused by uneven particle distribution and particle size. There is a linear correlation between MSC and SNV, which may lead to similar results of the two treatments. NM can unify the range of data, eliminate the influence of dimensions, and make the absolute value of numerical values become some kinds of relative value relationship, which is an effective way to simplify calculation and reduce numerical values. In similar studies, it can also be proved that NM has good spectral preprocessing effect [14]. This meant that the NM preprocessing treatment was suitable for the identification of pesticide residues in Hami melon.

In order to verify the classification effect of the tHBA-ELM model, different optimization strategies (GA, HBA) were used to optimize the ELM model and experiments were performed under the same dataset. After NM preprocessing, three ELM classification models based on different metaheuristic optimization algorithms have improved the classification effect and accuracy. Among them, the classification accuracy of NM-tHBA-ELM model was the highest, followed by the NM-HBA-ELM effect, and NM-GA-ELM effect was the worst. This may be because the introduction of adaptive t-distribution variation enhanced the diversity of population, which was beneficial to the algorithm to jump out of local optimization and improve the search speed.

Among single-class discrimination, Acetamiprid and Difenoconazole have the highest discrimination accuracy. A possible reason for this is that these two pesticides are stable, non-volatile, and the reflection ability for the spectral is strong [7,61]. The classification accuracy of Beta-cypermethrin was basically lower than that of the other four categories in the classification experiment of pesticide residues on the surface of Hami melon. A possible reason for this is that Beta-cypermethrin belongs to an agricultural pesticide, and its main component is cypermethrin, which is stable and not volatile in low temperature environments. However, when collecting hyperspectral images, the temperature in the dark box of HIS system will be slightly higher than the room temperature due to the energy excitation of halogen lamps. Therefore, when collecting Hami melon samples with Beta-cypermethrin on the surface, a small amount of surface pesticides may be heated and volatilized, resulting in poor classification effect compared with other pesticides.

Suitable preprocessing methods and meta-heuristic optimizers can effectively optimize the ELM model and thus improve the classification performance. A comparison of results from classification experiments on the identification of pesticide residue species on the surface of Hami melon based on different models reveals the superiority of NM-tHBA-ELM. Moreover, the fast convergence speed and good adaptation values further confirmed the effectiveness of our improvements on HBA. The results of the present study will help in the non-destructive detection of pesticide residues on the surfaces of other fruits and vegetables, thus effectively addressing the issue of food safety.

## 5. Conclusions

In this study, the tHBA-ELM model was successfully combined with SWIR-HSI in the spectral wavelength range of 1000–2500 nm, and a novel method for pesticide residue detection on Hami melon surface was proposed. The SWIR hyperspectral image was collected, and the spectral information of the sample was obtained with 50 × 50 pixel blocks as the region of interest. The classification effect of classical machine learning classification models (ELM, SVM, and PLS-DA) on Hami melon surface pesticide residues was compared, and ELM was selected as the benchmark model. Different preprocessing treatments were adopted to measure their impact on the ELM classification model, and NM preprocessing treatment was proven to have the best performance. In order to improve the accuracy of NM-ELM model, the tHBA optimization algorithm was proposed, and it was proven that the tHBA optimization algorithm had faster convergence speed and better optimization effect than HBA and GA. The overall accuracy, precision, sensitivity, and F1-score of the NM-tHBA-ELM model was 93.50%, 93.73%, 93.50%, and 0.9355, which were higher than other classification models. Overall, the results showed that tHBA-ELM combined with SWIR hyperspectral imaging can be used as a non-destructive and efficient method for detecting pesticide residues on the surface of Hami melon. In addition, this method can provide a reference for the detection of pesticide residues in other fruits and vegetables. Furthermore, the types of pesticide residues on the surface of Hami melon and the varieties of Hami melon are not the same. Some parameters of the model should be fine-tuned with changes of pesticides and Hami melon varieties. Future work will optimize the generalizability of the proposed method by spraying more kinds of pesticides and adding different varieties of Hami melon samples, as well as exploring the use of deep learning and HSI technology to complete the non-destructive detection of pesticide residues on the surface of Hami melon [62,63].

## Figures and Tables

**Table 1:** Classification performance of three classical machine learning models for pesticide residues.

Models	Class	Accuracy (%)	Precision (%)	Sensitivity (%)	F1-Score
ELM	All	79.50	80.29	79.50	0.7962
	None	75.00	78.95	75.00	0.7692
	Acetamiprid	82.50	89.19	82.50	0.8571
	Malathion	82.50	73.33	82.50	0.7765
	Difenoconazole	82.50	71.74	82.50	0.7674
	Beta-cypermethrin	75.00	88.24	75.00	0.8108
SVM	All	77.50	78.03	77.50	0.7760
	None	75.00	78.95	75.00	0.7692
	Acetamiprid	77.50	86.11	77.50	0.8158
	Malathion	75.00	78.95	75.00	0.7692
	Difenoconazole	80.00	68.09	80.00	0.7356
	Beta-cypermethrin	80.00	78.05	80.00	0.7901
PLS-DA	All	75.50	76.42	75.50	0.7555
	None	75.00	76.92	75.00	0.7595
	Acetamiprid	67.50	87.10	67.50	0.7606
	Malathion	82.50	71.74	82.50	0.7674
	Difenoconazole	77.50	67.39	77.50	0.7209
	Beta-cypermethrin	75.00	78.95	75.00	0.7692

**Table 2:** Influence of different preprocessing treatments on ELM model performance.

Models	Class	Accuracy (%)	Precision (%)	Sensitivity (%)	F1-Score
NM- ELM	All	82.00	82.07	82.00	0.8201
	None	85.00	79.07	85.00	0.8193
	Acetamiprid	90.00	92.31	90.00	0.9114
	Malathion	80.00	82.05	80.00	0.8101
	Difenoconazole	80.00	80.00	80.00	0.8000
	Beta-cypermethrin	75.00	76.92	75.00	0.7595
MSC-ELM	All	80.50	80.87	80.50	0.8062
	None	82.50	84.62	82.50	0.8354
	Acetamiprid	85.00	91.89	85.00	0.8831
	Malathion	82.50	78.57	82.50	0.8049
	Difenoconazole	75.00	69.77	75.00	0.7229
	Beta-cypermethrin	77.50	79.49	77.50	0.7848
SNV-ELM	All	80.00	80.08	80.00	0.8001
	None	82.50	84.62	82.50	0.8354
	Acetamiprid	82.50	84.62	82.50	0.8354
	Malathion	75.00	76.92	75.00	0.7595
	Difenoconazole	82.50	76.74	82.50	0.7952
	Beta-cypermethrin	77.50	77.50	77.50	0.7750

**Table 3:** Classification results of pesticide residues in Hami melon based on ELM model with different optimization algorithms.

Models	Class	Accuracy (%)	Precision (%)	Sensitivity (%)	F1-Score
NM- GA- ELM	All	88.50	88.72	88.50	0.8852
	None	90.00	92.31	90.00	0.9114
	Acetamiprid	95.00	90.48	95.00	0.9268
	Malathion	87.50	92.11	87.50	0.8974
	Difenoconazole	82.50	89.19	82.50	0.8571
	Beta-cypermethrin	87.50	79.55	87.50	0.8333
NM-HBA-ELM	All	90.50	90.66	90.50	0.9051
	None	95.00	88.37	95.00	0.9157
	Acetamiprid	92.50	94.87	92.50	0.9367
	Malathion	87.50	94.59	87.50	0.9091
	Difenoconazole	90.00	85.71	90.00	0.8780
	Beta-cypermethrin	87.50	89.74	87.50	0.8861
NM-tHBA-ELM	All	93.50	93.73	93.50	0.9355
	None	92.50	88.10	92.50	0.9024
	Acetamiprid	95.00	97.44	95.00	0.9620
	Malathion	92.50	97.37	92.50	0.9487
	Difenoconazole	95.00	88.37	95.00	0.9157
	Beta-cypermethrin	92.50	97.37	92.50	0.9487

## Data Availability

The data presented in this study are available on request from the corresponding author.
